# Dosage Compensation and the Distribution of Sex-Biased Gene Expression in *Drosophila*: Considerations and Genomic Constraints

**DOI:** 10.1007/s00239-016-9735-y

**Published:** 2016-04-08

**Authors:** Miguel Gallach, Esther Betrán

**Affiliations:** Center for Integrative Bioinformatics Vienna (CIBIV), Max F Perutz Laboratories (MFPL), University of Vienna and Medical University of Vienna, Dr. Bohrgasse 9, 1030 Vienna, Austria; Department of Biology, University of Texas at Arlington, Arlington, USA

**Keywords:** Dosage compensation, X chromosome, Sex-biased gene expression, *Drosophila*

## Abstract

Several studies in *Drosophila* have shown a paucity of male-biased genes (i.e., genes that express higher in males than in females) on the X chromosome. Dosage compensation (DC) is a regulatory mechanism of gene expression triggered in males that hypertranscribes the X-linked genes to the level of transcription in females. There are currently two different hypotheses about the effects of DC on the distribution of male-biased genes: (1) it might limit male-expression level, or (2) it might interfere with the male upregulation of gene expression. Here, we used previously published gene expression datasets to reevaluate both hypotheses and introduce a mutually exclusive prediction that helped us to reject the hypothesis that the paucity of male-biased genes in the X chromosome is due to a limit in the male-expression level. Our analysis also uncovers unanticipated details about how DC interferes with the genomic distribution of both, male-biased and female-biased genes. We suggest that DC actually interferes with female downregulation of gene expression and not male upregulation, as previously suggested.

## Introduction

In *Drosophila* (fruit flies), females carry two X chromosomes (XX) while males carry one X chromosome and one Y chromosome (XY). Because the Y chromosome is highly degenerated and nearly devoid of genes (Carvalho et al. 2009), males require a molecular system to compensate the hemizygosity at the X chromosome (Baker et al. 1994). Such a regulatory system of gene expression is known as dosage compensation (DC). DC is achieved in *Drosophila* males by a set of chromatin modifications on the X chromosome that enhance the processivity of the RNA polymerase during the transcription of the X-linked genes (Lucchesi et al. 2005; Larschan et al. 2011). These modifications are triggered by a ribonucleoprotein complex known as dosage compensation complex (DCC). How DC may affect the genome-wide distribution of male-biased genes is a matter of debate (Vicoso and Charlesworth 2006, 2009; Bachtrog et al. 2010; Vensko and Stone 2014; Huylmans and Parsch 2015).

The differential expression of genes between males and females is known as sex-biased gene expression. There are different ways for a gene to achieve sex-biased expression from an unbiased ancestral state. However, it has been shown that most male-biased gene expression that originated in the *D. melanogaster* lineage occurred by upregulation of gene expression in males (Connallon and Knowles 2005; Vicoso and Charlesworth 2009; Gallach and Betran, under review). According to some studies, the upregulation of gene expression in males as the main mechanism to evolve male-biased gene expression would be incompatible with DC (Vicoso and Charlesworth 2009; Bachtrog et al. 2010). Such a conflict would explain the paucity of male-biased genes on the X chromosome of *Drosophila* (a. k. a. demasculinization of the X chromosome; Parisi et al. 2003; Ranz et al. 2003; Zhang et al. 2007; Meisel et al. 2012; but see Meiklejohn and Presgraves 2012). Because DC is an X-specific phenomenon occurring in males, two hypotheses have been suggested about the influence of DC on the distribution of male-biased genes (Vicoso and Charlesworth 2009; Bachtrog et al. 2010). A DC hypothesis of the distribution of the male-biased genes was first suggested by Vicoso and Charlesworth (2006, 2009). The authors suggest that if there is a limit in gene expression level that can be attained, dosage compensated genes in males will be closer to such a limit than non-dosage compensated genes. Therefore, evolving male-biased gene expression by means of an increase in transcription rate should be harder for X-linked genes than for autosomal genes. We will refer to this hypothesis as the “DC limiting hypothesis.” An alternative hypothesis suggests that there is a direct “interference” of chromatin remodeling complexes and the DCC on the X chromosome, which impedes upregulation in males beyond that induced by DC (Bachtrog et al. 2010). We will refer to this hypothesis as the “DC interference hypothesis.” The DC limiting hypothesis predicts that there will be a deficit of male-biased genes on the X chromosome compared to the autosomes, and this deficit will be stronger for genes with high expression than for genes with low expression. The DC interference hypothesis predicts that male-biased genes will be scarce in regions bound by the DCC compared to unbound regions, regardless of the expression level. These predictions have not been tested yet.

In an attempt to better understand the constraints that DC imposes on changes in gene expression, we tested whether the distribution of male-biased genes across bound and unbound regions depends on the gene expression level. We also uncover unanticipated details about how DC interferes with the genomic distribution of both, male-biased and female-biased genes.

## Materials and Methods

### Data

We used the data from Bachtrog et al. (2010) as the main database for our analyses. The database included two gene expression profiles from whole adult (Parisi et al. 2003; Zhang et al. 2007), one from fly gonads (Parisi et al. 2003) and one from gonadectomized flies (Parisi et al. 2003), as well as the chromosomal coordinates of the DCC binding regions (*D. melanogaster* release 5.5). These four gene expression profiles were obtained using microarrays and the expression level measured as hybridization signal intensity. The DCC binding regions were previously identified from high-resolution ChIP-chip mapping data in MSL3 mutant male embryos. According to the original study, a gene was classified as “bound” if it overlaps with a DCC binding region and as “unbound” otherwise (Alekseyenko et al. 2006). High affinity sites for the DCC (Alekseyenko et al. 2008) were not considered in this study. We also included in our analysis RNA-sequencing (RNA-Seq) data from two recent studies, which measured gene expression in whole adult as reads per kilobase per million reads (RPKM; Daines et al. 2011) and fragments per kilobase per million reads (FPKM; Graveley et al. 2011). The RNA-Seq data were integrated in our database based on the FlyBase identifier (FBgn number) associated with each gene. We classified genes as male-biased and female-biased when the differences in expression level between males and females were significant at a false discovery rate of 5 %, as computed in the original studies. Otherwise, genes were classified as unbiased.

Data manipulation and statistical analysis were performed in R (http://www.r-project.org).

## Results

### DC Interferes with the Genomic Distribution of Male-Biased and Female-Biased Genes

**Fig. 1:**
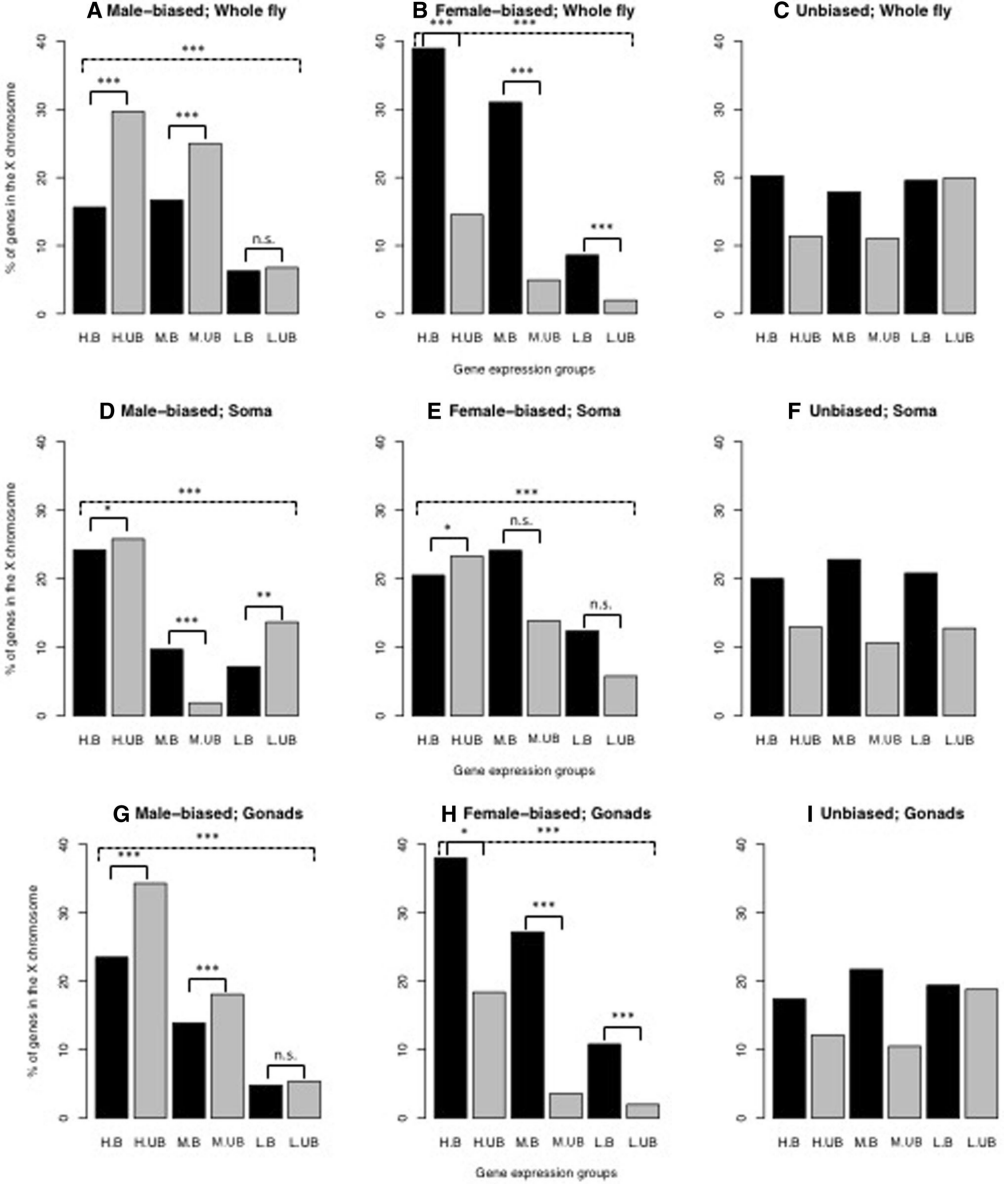
Percentages of genes with high, medium and low expression levels located in bound (*black*) and unbound regions (*gray*) of the X chromosome. Expression data correspond to whole flies (*first row* **a**–**c**), gonadectomized flies (*second row* **d**–**f**), and gonads (*third row* **g**–**i**). All dataset comparisons (*dashed lines*) and paired comparisons (*continuous lines*) were tested against the distribution of unbiased genes using a Chi squared test with 5 and 1 degrees of freedom, respectively. H, M and L refer to high, medium and low gene expression, respectively. B and UB refers to bound and unbound genes, respectively. *P* < 0.05 (*asterisk*), *P* < 0.01 (*double asterisk*), and *P* < 0.001 (*triple asterisk*). Data from Parisi et al. (2003)

We introduce a new prediction that helps us to distinguish between the DC limiting hypothesis and the DC interference hypothesis. According to the DC interference hypothesis, we should find a paucity of male-biased genes in bound regions compared to unbound regions, regardless of their expression level. According to the DC limiting hypothesis, due to the hypothetical transcriptional limit, male-biased genes expressing at high levels will be mainly located in unbound regions, but male-biased genes with medium or low expression levels should be equally distributed in bound and unbound regions. To contrast both hypotheses, we used gene expression data from whole adult flies (Parisi et al. 2003) and classified the genes into three groups of equal size according to their expression level (low, medium, and high expression), as in Vicoso and Charlesworth (2009). For this and other statistical analysis below, Chi square tests were applied to test for independence between DCC binding regions and sex-biased genes. Percentages of unbiased genes located in bound and unbound regions were used to calculate departures from the expected numbers. Our analysis shows a significant underrepresentation of male-biased genes in bound regions regardless of their expression level (*X*^2^ = 126.35; *df* = 5; *P* = 1.42 × 10^−25^), supporting the DC interference hypothesis (Fig. 1a).

Typical chromatin modifications associated with DC, as well as chromatin modifier proteins interacting with the DCC in males, are also enriched on the X chromosome in females, indicating that the chromatin structure of the X chromosomes also differs from the autosomes in this sex (Jin et al. 2000; Kind et al. 2008; Zhang and Oliver 2010; Sala et al. 2011; Brown and Bachtrog 2014). This prompted us to investigate whether DC also “interferes” with the distribution of female-biased genes. To do so, we classified female-biased genes into three groups of equal size according to their expression level, as we did for male-biased genes. Interestingly, our analysis reveals that female-biased genes are significantly enriched in bound regions, regardless of their expression level (*X*^2^ = 162.17; *df* = 5; *P* = 3.42 × 10^−33^; Fig. 1b). Therefore, DC seems to interfere with the distribution of female-biased genes, but in the opposite direction of that found for male-biased genes. This effect is observable in the supplementary information published by Bachtrog and colleagues, yet overlooked in the text (Bachtrog et al. 2010).

We extended our analysis to three additional published data sets in which whole adult were used to measure gene expression level (Table 1). In one of the studies (Zhang et al. 2007; “Zhang” in Table 1), gene expression was measured with microarrays and in the other two (Daines et al. 2011; Graveley et al. 2011; “Daines” and “Graveley” in Table 1), by RNA-Seq. In all cases, we found a significant underrepresentation in bound regions of male-biased genes and an overrepresentation of female-biased genes in bound regions. Therefore, this pattern is reproducible across biological replicates and platforms used to quantify gene expression.

**Table 1:** Number and fraction (in brackets) of male-biased and female-biased genes located in bound and unbound regions

Bias	Region	Parisi Whole fly	Zhang Whole fly	Daines Whole fly	Graveley Whole fly	Average Whole fly
Male	Bound Unbound Chi squared *P* value	74 (0.39) 118 (0.61) 29.84 4.69 × 10^−8^	55 (0.26) 154 (0.74) 69.48 <2.20 × 10^−16^	47 (0.24) 153 (0.77) 160.98 <2.20 × 10^−16^	40 (0.22) 146 (0.78) 129.46 <2.20 × 10^−16^	54 (0.27) 143 (0.73) 87.18 <2.20 × 10^−16^
Female	Bound Unbound Chi squared *P* value	238 (0.79) 65 (0.31) 52.52 4.27 × 10^−13^	73 (0.66) 37 (0.34) 5.74 0.02	210 (0.8) 55 (0.2) 20.72 5.32 × 10^−6^	184 (0.74) 63 (0.26) 16.37 5.22 × 10^−5^	176 (0.76) 55 (0.34) 25.23 5.09 × 10^−7^
Unbiased	Bound Unbound	339 (0.58) 249 (0.42)	764 (0.55) 621 (0.45)	730 (0.66) 374 (0.34)	846 (0.62) 523 (0.38)	670 (0.60) 442 (0.4)

The percentages of unbiased genes located in bound and unbound regions were used to calculate the expected number of genes located in each region. *P* values were calculated for the Chi squared test values with 1 degree of freedom. Parisi: data from Parisi et al. (2003); Zhang: data from Zhang et al. (2007); Daines: data from Daines et al. (2011); Graveley: data from Graveley et al. (2011)

To gain a better insight about the differences between male-biased genes and female-biased genes, we extended our analysis to published gene expression data from gonads and gonadectomized adult flies (Parisi et al. 2003). Consistently, we found that, in gonads, there is an underrepresentation of male-biased genes in bound regions and an overrepresentation of female-biased genes in those regions, regardless of the expression level (*X*^2^ = 118.34; *df* = 5; *P* = 7.03 × 10^−24^ for male-biased genes and *X*^2^ = 130.48; *df* = 5; *P* = 1.88 × 10^−26^ for female-biased genes; Fig. 1g, h). However, this pattern was not observed in somatic tissues (i.e., gonadectomized flies; Fig. 1d, e), suggesting that a different selective pressure for sex-biased genes is at work in gonads compared to the somatic tissues. The difference between gonads (Fig. 1g, h) and gonadectomized samples (Fig. 1d, e) suggests that, even if excision of germline tissue from the adult carcass was incomplete, this contamination is not enough as to replicate the pattern found in gonads. Alternatively, the absence of a clear pattern in gonadectomized flies may reflect the heterogeneity among somatic tissues in their sex bias expression patterns (Meisel et al. 2012; Huylmans and Parsch 2015).

### Dosage Compensation Does Not Impede Further Upregulation of Genes in Males

**Fig. 2:**
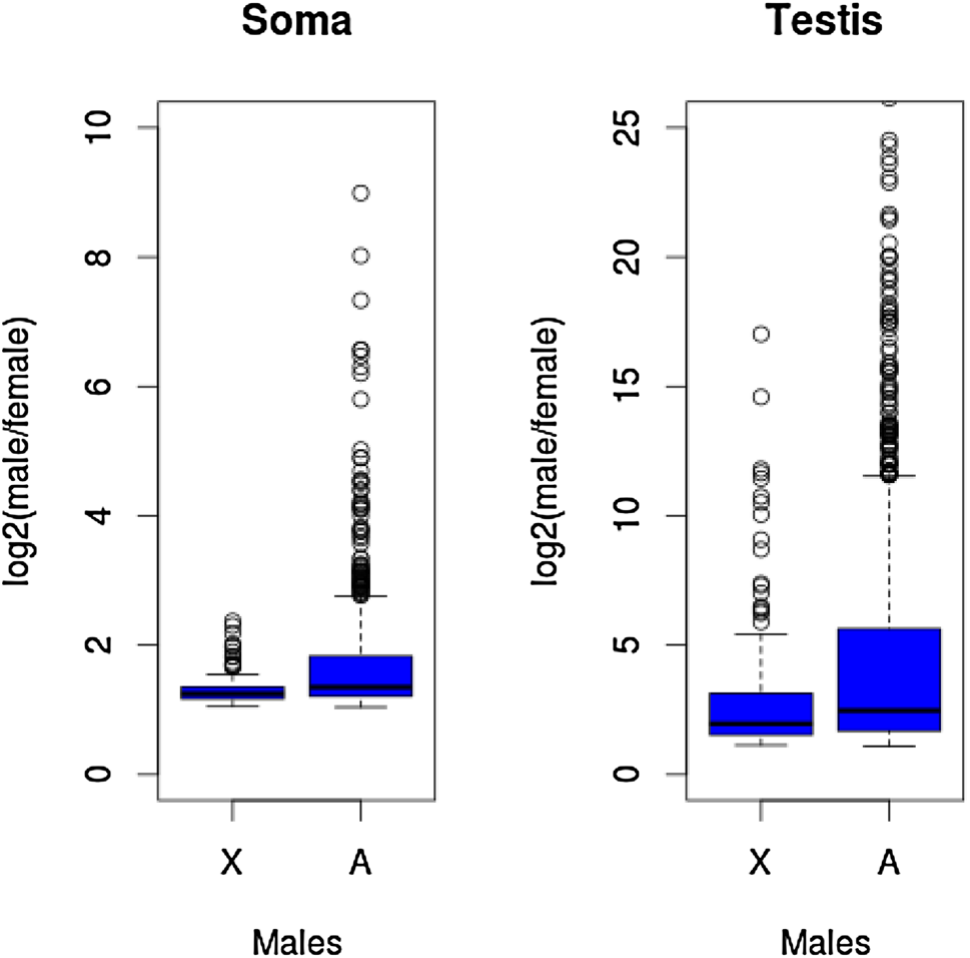
Male/female expression ratio for X-linked genes and autosomal genes. Only male-biased genes are considered. Wilcoxon rank-sum test was performed for each pair of data. *P* < 0.001, each

**Fig. 3:**
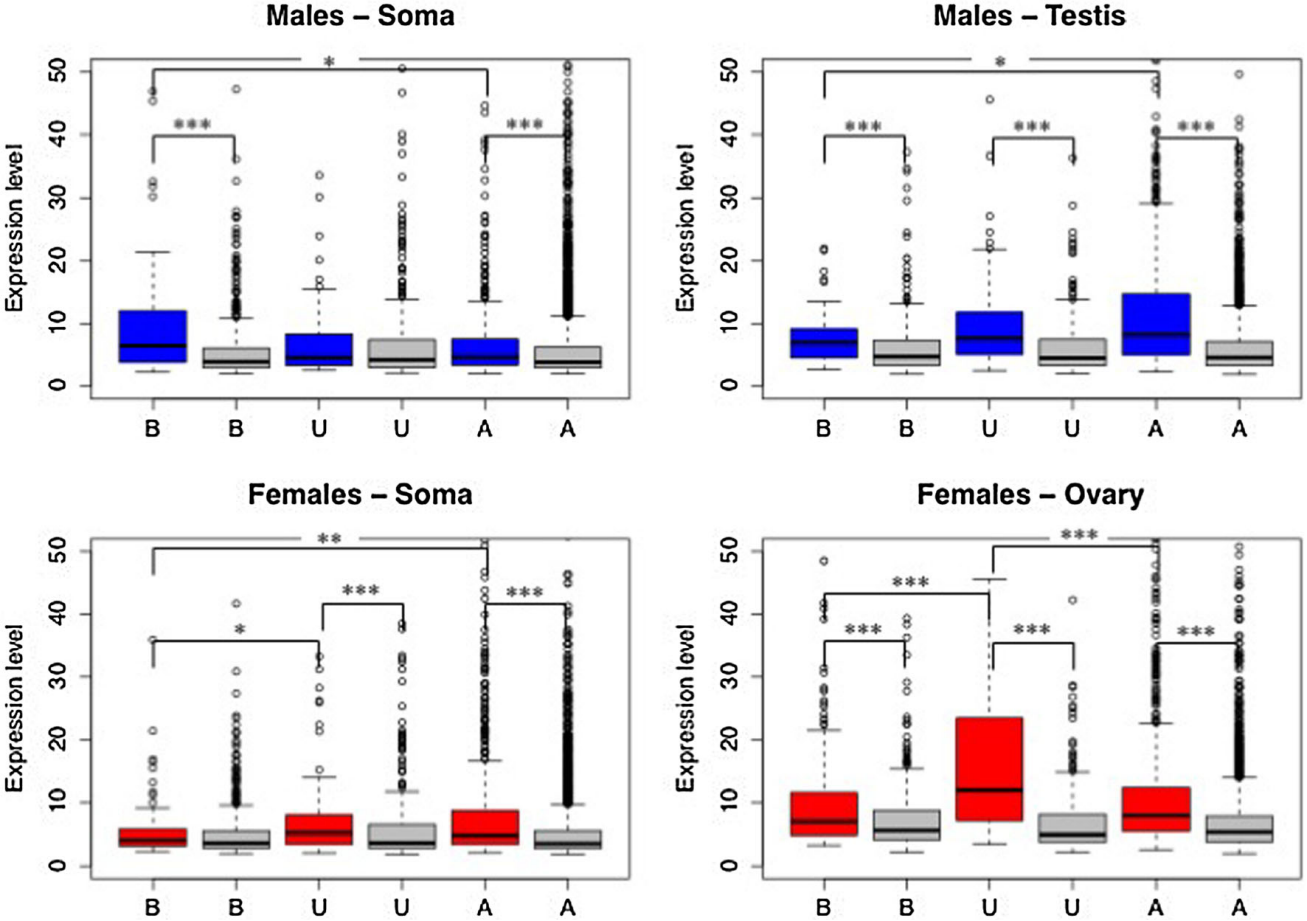
Expression level of male-biased genes (*blue*), female-biased genes (*red*) and unbiased genes (*gray*) located in bound regions (B), unbound regions (U) and autosomes (A). Paired comparisons were tested using the Wilcoxon rank-sum test. Only significant comparisons are indicated. *P* < 0.05 (*asterisk*), *P* < 0.01 (*double asterisk*), and *P* < 0.001 (*triple asterisk*) (Color figure online)

According to both DC hypotheses for the distribution of male-biased genes, impeding upregulation of gene expression in males would explain why the male bias level (i.e., the male/female expression ratio) is significantly lower for X-linked genes compared to autosomal genes (Bachtrog et al. 2010; Assis et al. 2012; Fig. 2). If DC constrained the further upregulation of gene expression above a certain limit, then we would expect male-biased genes in DCC binding regions to be expressed in males at lower levels than in unbound regions and autosomes. We used data from gonads and gonadectomized flies (Parisi et al. 2003) to test this hypothesis. Contrary to this prediction, the expression level of male-biased genes in somatic tissues (where there is DC) is higher in bound regions than in autosomes (*P* < 0.001, Wilcoxon rank-sum test; Fig. 3). The expression level of male-biased genes in testis (where the DCC is not expressed) is lower in bound regions than in autosomes (*P* < 0.001, Wilcoxon rank-sum test; Fig. 3). Meiotic sex chromosome inactivation (Vibranovski et al. 2009) or a similar mechanism (Meiklejohn et al. 2011; Kemkemer et al. 2014) as well as the absence of DC in testis (Meiklejohn et al. 2011) could explain why X-linked male-biased genes express lower in testes compared to autosomes. However, this is not likely the case because unbiased genes located in the same chromosomes do not show the same pattern as male-biased genes (Fig. 3).

### High Male Bias Level of Gene Expression Requires Low Gene Expression in Females

**Fig. 4:**
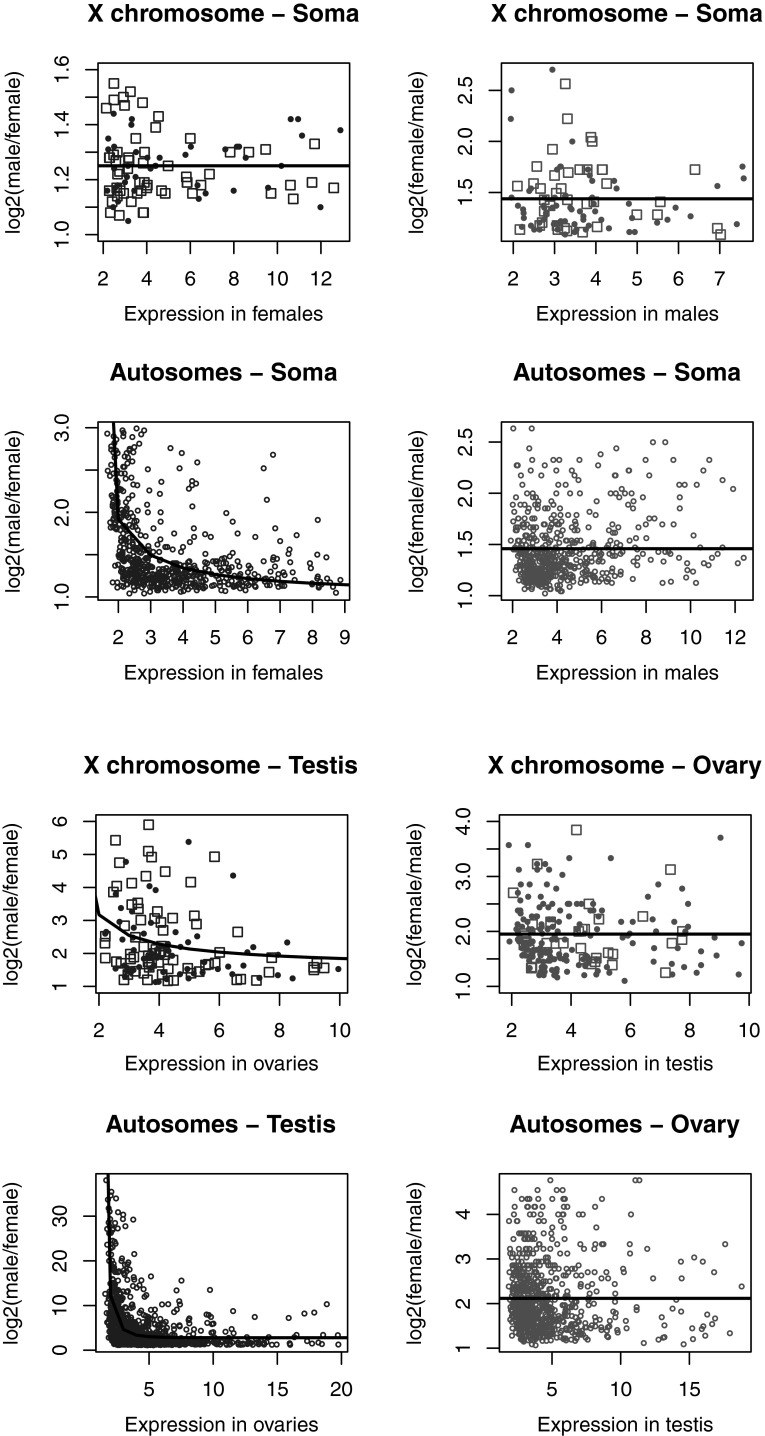
Correlation between the sex bias level in the sex with higher expression and the gene expression level in the opposite sex. In gonadectomized flies (soma, *first four panels*) only male-biased genes located in the autosomes correlate with the expression level in females: the lower the expression level in females, the higher the difference between males and females. This effect is however minimal compared to gonads (*last four panels*), where genes linked to the autosomes that express low in females reach the highest male-biased levels. In addition, it can be seen that the effect of the gene expression level in the opposite sex over the sex bias level is insignificant for genes bound (*dots*) and unbound by the DCC (*open squares*). *Blue dots* male-biased genes. *Red dots* female-biased genes. *Black lines* correspond to the fitted functions (Color figure online)

Our analyses do not support the hypothesis that DC constrains the upregulation of male-biased genes. Then, why is the male bias level significantly lower for X-linked genes compared to autosomal genes (Bachtrog et al. 2010; Assis et al. 2012; Fig. 2)? The alternative explanation is that DC constrains the downregulation of gene expression in females. In Fig. 4, we plot the sex bias level in the sex with higher expression as a function of the gene expression level in the other sex. Altogether, the data show that male-biased genes can reach very high sex bias levels, but this only happens when their expression in females is low, and this especially occurs for genes located on the autosomes. In contrast, female bias level is not only lower than that of male-biased genes, but also does not require low expression level in males.

## Discussion

Our analyses indicate that male-biased genes are expressed higher in regions bound by the DCC than in unbound regions and in autosomes in somatic tissues (where DC takes place). This observation is incompatible with the hypothesis that DC and other chromatin remodeling proteins impede the upregulation of gene expression (Vicoso and Charlesworth 2009; Bachtrog et al. 2010). Is there any evidence supporting the hypothesis that chromatin remodeling complexes interacting with the DCC impede the upregulation of gene expression in males? Some chromatin remodeling proteins, such as ISWI, the ATAC complex, and SU(VAR)3-7, do certainly have specific roles in the X chromosomes or even interact genetically with DC (Corona et al. 2007; Carré et al. 2008; Spierer et al. 2008; Sala et al. 2011). If these proteins impeded upregulation of dosage compensated genes, we would expect dosage compensated genes to be upregulated in null flies for *iswi*, *atac* of *su(var)3*-*7*. However, mutations in these genes do not upregulate X-linked genes or even cause a generalized misregulation of the X-linked genes. Therefore, to our knowledge, there is no evidence to suggest that these proteins impede upregulation of X-linked genes in males (Bachtrog et al. 2010).

Chromatin modifications associated with DC and open chromatin structure still persist in females (Jin et al. 2000; Kind et al. 2008; Zhang and Oliver 2010; Sala et al. 2011; Brown and Bachtrog 2014). One important consequence that comes out from this feature is that silencing or downregulating gene expression may be a harder task for X-linked genes in females than for autosomal genes (Zhang and Oliver 2010). Consistently, autosomal testis-biased genes, as well as other autosomal tissue-specific genes, are enriched with repressors of gene expression in other tissues, while in the case of the X-linked genes, this trend is reduced or reversed in favor of activators of gene expression (Mikhaylova and Nurminsky 2011). In other words, because of DC, the X chromosome most likely provides an inadequate environment for genes that need repression in some tissues or females (Zhang and Oliver 2010; Mikhaylova and Nurminsky 2011). This simple model may explain why high male bias levels mainly occur on the autosomes or far from DCC (Bachtrog et al. 2010), where gene expression can be easily downregulated in females. This pattern is especially strong for testis-biased genes (Fig. 4), because testis represents the largest group of tissue-specific genes in *Drosophila* (Chintapalli et al. 2007) and it might extend to non-coding genes as well (Gao et al. 2014).
